# Supplementary material for: Impedance-derived phase angle is associated with muscle mass, strength, quality of life, and clinical outcomes in maintenance hemodialysis patients
Source: PLoS One. 2022 Jan 12;17(1):e0261070. doi: 10.1371/journal.pone.0261070 (PMC8754345; doi:10.1371/journal.pone.0261070)
Supplement: S1 Table — (DOCX) [file pone.0261070.s001.docx]

**S1 Table. Correlation between phase angle and quality-of-life scales**

|  | *r* | *P*-value |
| --- | --- | --- |
| PCS | 0.245 | 0.025 |
| MCS | 0.078 | 0.486 |
| KDCS | 0.377 | 0.123 |
| BDI | –0.278 | 0.011 |
| BAI | –0.127 | 0.027 |

Abbreviations: PCS, physical component scale; MCS, mental component scale; KDCS, kidney disease component scale; BDI, Beck Depression Inventory; BAI, Beck Anxiety Inventory.
