# Supplementary material for: Impedance-derived phase angle is associated with muscle mass, strength, quality of life, and clinical outcomes in maintenance hemodialysis patients
Source: PLoS One. 2022 Jan 12;17(1):e0261070. doi: 10.1371/journal.pone.0261070 (PMC8754345; doi:10.1371/journal.pone.0261070)
Supplement: S2 Table — (DOCX) [file pone.0261070.s002.docx]

**S2 Table. Correlation between phase angle and various indices according to age**

|  | **<57 years** | | |  | | | **≥ 57 years** | | |
| --- | --- | --- | --- | --- | --- | --- | --- | --- | --- |
|  | **Univariate** | | **Multivariate** | |  | **Univariate** | | **Multivariate** | |
|  | ***r*** | ***P*-value** | ***r*** | ***P*-value** |  | ***r*** | ***P*-value** | ***r*** | ***P*-value** |
| Handgrip strength (kg) | 0.631 | <0.001 | 0.520 | 0.001 |  | 0.293 | 0.060 | 0.173 | 0.285 |
| SGA score | 0.578 | <0.001 | 0.700 | <0.001 |  | 0.246 | 0.116 | 0.165 | 0.309 |
| Serum albumin (mg/dL) | 0.066 | 0.684 | 0.081 | 0.625 |  | –0.208 | 0.185 | –0.187 | 0.247 |
| Body mass index | 0.239 | 0.133 | 0.346 | 0.031 |  | 0.056 | 0.725 | 0.075 | 0.645 |
| TMA/Ht^2^ | 0.631 | <0.001 | 0.628 | <0.001 |  | 0.349 | 0.023 | 0.285 | 0.074 |
| Gait speed | 0.664 | <0.001 | 0.593 | <0.001 |  | 0.234 | 0.136 | 0.192 | 0.235 |
| SPPB | 0.389 | 0.012 | 0.341 | 0.034 |  | 0.141 | 0.374 | 0.144 | 0.377 |
| 5STS | –0.489 | 0.001 | –0.522 | 0.001 |  | –0.272 | 0.082 | –0.206 | 0.203 |
| STS30 | 0.567 | <0.001 | 0.548 | <0.001 |  | 0.261 | 0.095 | 0.221 | 0.170 |
| 6-MWT | 0.524 | <0.001 | 0.432 | 0.006 |  | 0.102 | 0.519 | 0.098 | 0.547 |
| Timed up-and-go test | –0.487 | 0.001 | –0.437 | 0.005 |  | –0.181 | 0.251 | –0.180 | 0.268 |

Correlations were analyzed using Pearson’s correlation for univariate analysis and partial correlation for multivariate analysis. The results of multivariate analysis were adjusted for sex and presence of diabetes mellitus.

Abbreviations: *r*, correlation coefficient; SGA, subjective global assessment; TMA/Ht^2^, thigh muscle area per height squared; SPPB, Short Physical Performance Battery; 5STS, five times sit-to-stand test; STS30, 30-s sit-to-stand test; 6-MWT, 6-min walk test.
