# Supplementary material for: Impedance-derived phase angle is associated with muscle mass, strength, quality of life, and clinical outcomes in maintenance hemodialysis patients
Source: PLoS One. 2022 Jan 12;17(1):e0261070. doi: 10.1371/journal.pone.0261070 (PMC8754345; doi:10.1371/journal.pone.0261070)
Supplement: S3 Table — (DOCX) [file pone.0261070.s003.docx]

**S3 Table. Correlation between phase angle and various indices according to sex**

|  | **Men** | | |  | | | **Women** | | |
| --- | --- | --- | --- | --- | --- | --- | --- | --- | --- |
|  | **Univariate** | | **Multivariate** | |  | **Univariate** | | **Multivariate** | |
|  | ***r*** | ***P*-value** | ***r*** | ***P*-value** |  | ***r*** | ***P*-value** | ***r*** | ***P*-value** |
| Handgrip strength (kg) | 0.448 | 0.003 | 0.357 | 0.022 |  | 0.301 | 0.059 | 0.212 | 0.202 |
| SGA score | 0.288 | 0.061 | 0.245 | 0.123 |  | 0.648 | <0.001 | 0.654 | <0.001 |
| Serum albumin (mg/dL) | 0.024 | 0.879 | –0.127 | 0.430 |  | –0.154 | 0.341 | –0.177 | 0.288 |
| Body mass index | 0.195 | 0.211 | 0.193 | 0.228 |  | 0.154 | 0.344 | 0.277 | 0.170 |
| TMA/Ht^2^ | 0.581 | <0.001 | 0.518 | 0.001 |  | 0.275 | 0.086 | 0.302 | 0.065 |
| Gait speed | 0.383 | 0.011 | 0.275 | 0.082 |  | 0.482 | 0.002 | 0.423 | 0.008 |
| SPPB | 0.238 | 0.124 | 0.138 | 0.388 |  | 0.362 | 0.022 | 0.270 | 0.101 |
| 5STS | –0.463 | 0.002 | –0.449 | 0.003 |  | –0.307 | 0.054 | –0.218 | 0.190 |
| STS30 | 0.486 | 0.001 | 0.422 | 0.006 |  | 0.329 | 0.038 | 0.246 | 0.137 |
| 6-MWT | 0.249 | 0.107 | 0.108 | 0.500 |  | 0.396 | 0.011 | 0.301 | 0.067 |
| Timed up-and-go test | –0.334 | 0.029 | –0.232 | 0.144 |  | –0.330 | 0.038 | –0.205 | 0.218 |

Correlations were analyzed using Pearson’s correlation for univariate analysis and partial correlation for multivariate analysis. The results of multivariate analysis were adjusted for age and presence of diabetes mellitus.

Abbreviations: *r*, correlation coefficient; SGA, subjective global assessment; TMA/Ht^2^, thigh muscle area per height squared; SPPB, Short Physical Performance Battery; 5STS, five times sit-to-stand test; STS30, 30-s sit-to-stand test; 6-MWT, 6-min walk test.
