# Supplementary material for: Impedance-derived phase angle is associated with muscle mass, strength, quality of life, and clinical outcomes in maintenance hemodialysis patients
Source: PLoS One. 2022 Jan 12;17(1):e0261070. doi: 10.1371/journal.pone.0261070 (PMC8754345; doi:10.1371/journal.pone.0261070)
Supplement: S4 Table — (DOCX) [file pone.0261070.s004.docx]

**S4 Table. Correlation between phase angle and various indices according to presence of diabetes mellitus**

|  | **Non-diabetes mellitus** | | |  | | | **Diabetes mellitus** | | |
| --- | --- | --- | --- | --- | --- | --- | --- | --- | --- |
|  | **Univariate** | | **Multivariate** | |  | **Univariate** | | **Multivariate** | |
|  | ***r*** | ***P*-value** | ***r*** | ***P*-value** |  | ***r*** | ***P*-value** | ***r*** | ***P*-value** |
| Handgrip strength (kg) | 0.568 | <0.001 | 0.406 | 0.013 |  | 0.336 | 0.026 | 0.200 | 0.205 |
| SGA score | 0.664 | <0.001 | 0.594 | <0.001 |  | 0.131 | 0.395 | 0.110 | 0.487 |
| Serum albumin (mg/dL) | 0.288 | 0.076 | 0.154 | 0.363 |  | –0.437 | 0.003 | –0.463 | 0.002 |
| Body mass index | 0.356 | 0.026 | 0.381 | 0.020 |  | 0.080 | 0.606 | 0.099 | 0.532 |
| TMA/Ht^2^ | 0.761 | <0.001 | 0.701 | <0.001 |  | 0.260 | 0.089 | 0.217 | 0.168 |
| Gait speed | 0.620 | <0.001 | 0.493 | 0.002 |  | 0.284 | 0.062 | 0.202 | 0.201 |
| SPPB | 0.466 | 0.003 | 0.355 | 0.031 |  | 0.142 | 0.359 | 0.136 | 0.392 |
| 5STS | –0.525 | 0.001 | –0.370 | 0.024 |  | –0.256 | 0.093 | –0.237 | 0.130 |
| STS30 | 0.566 | <0.001 | 0.411 | 0.011 |  | 0.264 | 0.083 | 0.258 | 0.100 |
| 6-MWT | 0.483 | 0.002 | 0.344 | 0.037 |  | 0.137 | 0.375 | 0.062 | 0.696 |
| Timed up-and-go test | –0.480 | 0.002 | –0.366 | 0.026 |  | –0.149 | 0.335 | –0.109 | 0.492 |

Correlations were analyzed using Pearson’s correlation for univariate analysis and partial correlation for multivariate analysis. The results of multivariate analysis were adjusted for age and sex.

Abbreviations: *r*, correlation coefficient; SGA, subjective global assessment; TMA/Ht^2^, thigh muscle area per height squared; SPPB, Short Physical Performance Battery; 5STS, five times sit-to-stand test; STS30, 30-s sit-to-stand test; 6-MWT, 6-min walk test.
